# The Effect of HIV Self-Testing Delivery Models on Female Sex Workers’ Sexual Behaviors: A Randomized Controlled Trial in Urban Uganda

**DOI:** 10.1007/s10461-019-02393-z

**Published:** 2019-01-16

**Authors:** Katrina F. Ortblad, Daniel Kibuuka Musoke, Thomson Ngabirano, Aidah Nakitende, Guy Harling, Jessica E. Haberer, Margaret McConnell, Joshua A. Salomon, Catherine E. Oldenburg, Till Bärnighausen

**Affiliations:** 1 Department of Global Health, University of Washington, 908 Jefferson St, 12th Floor, Seattle, WA 98104 USA; 2 International Research Consortium, Kampala, Uganda; 3 Uganda Health Marketing Group, Kampala, Uganda; 4 Institute for Global Health, University College London, London, UK; 5 Department of General Internal Medicine, Massachusetts General Hospital Global Health, Boston, MA USA; 6 Department of Global Health and Population, Harvard T.H. Chan School of Public Health, Boston, MA USA; 7 Department of Medicine, Stanford University, Stanford, CA USA; 8 Francis I. Proctor Foundation, University of California San Francisco, San Francisco, CA USA; 9 Department of Ophthalmology, University of California, San Francisco, San Francisco, CA USA; 10 Department of Epidemiology & Biostatistics, University of California, San Francisco, CA USA; 11 Heidelberg Institute of Public Health, University of Heidelberg, Heidelberg, Germany; 12 Africa Health Research Institute, Somkhele, KwaZulu-Natal South Africa

**Keywords:** HIV self-testing, Sexual behaviors, Condom use, Female sex workers, Uganda

## Abstract

HIV self-testing increases recent and frequent HIV testing among female sex workers (FSWs) in urban Uganda. Using results from a randomized controlled trial, we aim to establish the effect of HIV self-testing delivery models on FSWs’ sexual behaviors in this setting. Clusters of one peer educator and eight participants were 1:1:1 randomized to: (1) direct provision of an HIV self-test, (2) provision of a coupon for facility collection of an HIV self-test, or (3) referral to standard-of-care HIV testing services. Sexual behaviors were self-reported at 1 and 4 months. From October to November 2016, 960 participants were enrolled and randomized. At 4 months, there were no statistically significant differences in participants’ sexual behaviors, including inconsistent condom use, across study arms. We do not find any changes in sexual risk-taking among FSWs in response to the delivery of HIV self-tests. Routine policies for HIV self-testing are likely a behaviorally safe component of comprehensive HIV prevention strategies.

## Introduction

HIV self-testing has been proven to increase HIV testing in diverse populations and settings [1–10]. HIV self-testing may be particularly beneficial for female sex workers (FSWs), who—according to the World Health Organization (WHO)—should test for HIV every 3 months, because they are at high risk of HIV acquisition [11]. Among FSWs in urban Uganda, the delivery of HIV self-tests increased recent and frequent HIV testing [7], suggesting that self-testing can overcome some of the barriers to HIV testing that FSWs face, such as facility hours [12], stigma and discrimination by healthcare providers [12–15], and transportation costs [16]. How HIV self-tests were delivered to Ugandan FSWs additionally affected how often they tested: FSWs tested for HIV more frequently when self-tests were delivered to them directly by peer educators (‘direct provision’) versus when they had to collect self-tests from a healthcare facility (‘facility collection’) [7].

Other behaviors, besides HIV testing, that influence FSWs’ risk of HIV transmission might also be affected by HIV self-tests [17–19]—and affected differentially depending on how HIV self-tests are delivered. When FSWs receive an HIV self-test directly from a peer educator instead of from a healthcare facility, they miss the opportunity to interact with a healthcare provider and receive services (e.g., counseling) or items (e.g., condoms) available at healthcare facilities. When FSWs pick-up HIV self-tests a healthcare facility, they might collect condoms and other free items, but still forgo counseling services. Reduced uptake of counseling services and reduced access to free condoms might increase sexual behaviors associated with HIV risk among FSWs. Conversely, it is also plausible that HIV self-testing approaches decrease FSWs’ HIV risk-related sexual behaviors. For instance, HIV self-testing may increase FSWs’ feeling of control and self-efficacy regarding HIV prevention, which may lead to greater sexual negotiation power and reduced risk behaviors [20, 21].

While there have been few studies to date on the effect of HIV self-testing on sexual behaviors, those that exist suggest that HIV self-testing may reduce sexual behaviors associated with HIV risk [17–19]. Among men who have sex with men in high income settings, the availability of HIV self-tests made men more selective with whom they had sex [17, 18]. Among FSWs in Zambian transit towns, HIV self-testing significantly reduced FSWs’ number of client and non-client sexual partners compared to standard testing services, and this effect was greater among FSWs who received HIV self-tests directly from a peer educator compare to those who went to a healthcare facility to collect HIV self-tests [19].

Using data from a three-arm HIV self-testing randomized controlled trial among FSWs in urban Uganda [7], we measure the effect of direct provision of and facility collection of HIV self-tests on FSWs’ sexual behaviors with client and non-client partners. Additionally we outline pathways through which the two different HIV self-test delivery models might affect FSWs’ sexual behaviors (e.g., empowerment, hope, communication of HIV status, confidence in HIV status) and measure the effect of the interventions on these pathways. Understanding the effect of different HIV self-testing health systems delivery models on FSWs’ sexual behaviors is important because many sub-Saharan African governments with generalized HIV epidemics consider FSWs a priority population for HIV prevention interventions [22] and are in the midst of rolling out HIV self-testing [23].

## Methods

### Ethics Statement

This study received Ethical Approval from the Institutional Review Board at the Harvard T.H. Chan School of Public Health and the Mildmay Uganda Research and Ethics Committee. All participants provided written informed consent.

### Participants and Study Setting

Between October to November 2016, FSWs in Kampala, Uganda were enrolled in a three-arm HIV self-testing cluster randomized controlled trial designed to measure the effect of different HIV self-testing delivery models on recent and frequent HIV testing (clinicaltrials.gov: NCT02846402). Complete methods for this study have been previously reported [7]. Participants were recruited by peer educators. Peer educators were referred by leaders from Kampala-based FSW peer organizations and clinics affiliated with Uganda’s Most at Risk Population Initiative. All peer educators completed a 2-day training prior to participant enrollment. Eligible participants were: 18 years or older, reported the exchange of sex for money or goods in the past month, did not know their HIV status or reported being HIV-negative and not having recently tested for HIV (past 3 months), and were Kampala-based. Research assistants called potential participants for an initial eligibility screening. They then invited potential participants for a more detailed in-person eligibility assessment, which was followed by an invitation to participate in this trial.

### Study Design

Peer educator–participant groups (one peer educator, eight participants) were randomized to: (1) *direct provision* of HIV self-tests, (2) provision of a coupon for healthcare *facility collection* of HIV self-tests, and (3) referral to *standard*-*of*-*care* HIV testing services. The randomization list was developed by the author CEO in R Studio (Version 3.3.1, The R Foundation for Statistical Computing, Vienna, Austria) in random blocks of 3, 6, and 9. Peer educator–participant group study assignments were placed in opaque, sealed envelopes that were opened by peer educator and research assistant, neither of whom knew the group assignment beforehand [7].

### Interventions

Over the duration of the study, participants completed four peer educator visits: 0, 0.5, 1.5, and 3 months after randomization. At these visits all participants received condoms, information on HIV prevention, and were encouraged to test for HIV at any standard testing facility. Participants in the HIV self-testing intervention arms additionally received one HIV self-test (OraQuick Rapid HIV-1/2 Antibody Test, OraSure Technologies, Bethlehem, PA) or one coupon, exchangeable for an HIV self-test at 10 participating private health facilities throughout Kampala, at the first and fourth peer educator visit [7]. HIV self-tests were distributed to participants in the intervention arms 3 months apart because this is the WHO’s recommended frequency of HIV testing for FSWs [24]. The interim peer educator visits were designed to keep peers and participants engaged in the study and prevent loss to follow-up.

### Assessments

Participants completed three quantitative assessments: a baseline assessment and two follow-up assessments. The baseline assessment occurred at participant enrollment, before randomization and distribution of HIV self-tests to participants in the intervention arms. The follow-up assessments occurred 1 and 4 months after the first peer educator visit. Socio-demographic characteristics were collected at baseline. Information related to recent HIV testing, including self-reported test results, and sexual behaviors with client and non-client partners was collected at all assessments. Research assistants collected the electronic data (CommCare, Dimagi, Inc., Cambridge, MA) in face-to-care interviews at private locations selected by participants (e.g. empty bar, home, etc.). Participants received 16,500 Uganda Shillings (~ 5 USD) upon completion of each assessment as compensation for their time.

### Sexual Behavior Outcomes

We measured four sexual behaviors outcomes: the average number of clients per night, inconsistent condom use with clients, the number of non-clients sexual partners in the past month, and inconsistent condom use with non-client sexual partners in the past month. Participants were asked to self-report the number of sexual clients they have on an average working night and the number of these with which they use condoms. Inconsistent condom use with clients was defined as not using a condom with at least one of these clients. Participants were additionally asked the number of sexual partners they had who were not clients in the past month and the number of these with whom they ever used condoms. Inconsistent condom use with non-clients was defined as never using a condom with at least one of these non-client sexual partners in the past month. Repeat clients and non-client sexual partners and condom use with these sexual partners are incorporated in these measures. If participants had a regular client with whom they do not use condoms, their condom use with clients was categorized as inconsistent. Similarly, if participants had a husband or boyfriend with whom they never use condoms, their condom use with non-clients was also be categorized as inconsistent. All sexual behavior outcomes were pre-specified for secondary analysis [25].

### Pathways for Sexual Behaviors

**Fig. 1:**
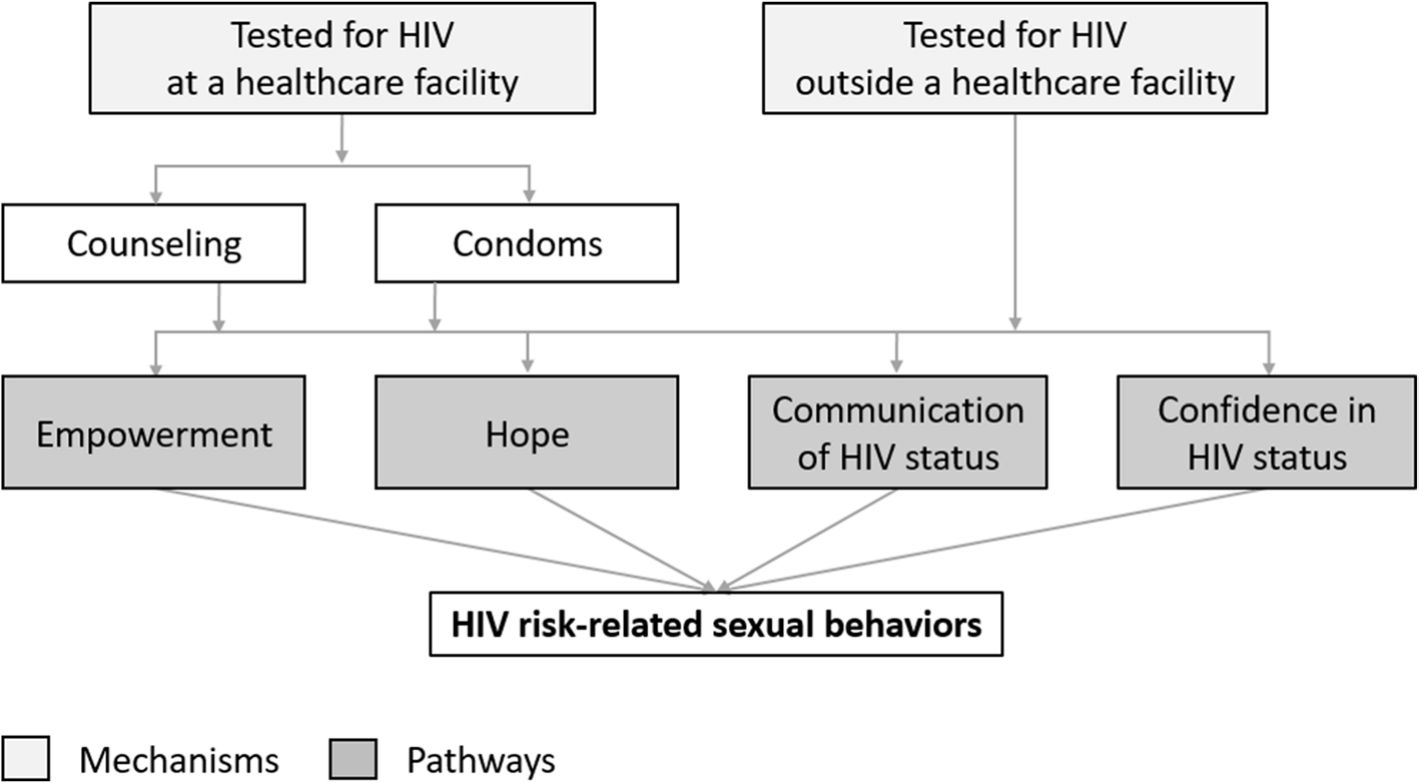
Pathways upstream from FSWs’ HIV risk-related sexual behaviors that may be affected by the different HIV self-testing delivery models. Pathway variables are depicted in dark gray boxes, while the mechanisms that might influence these pathways are depicted in light gray boxes

We additionally measured the effect of the different HIV self-testing delivery models on upstream pathways that we hypothesized might affect FSWs’ sexual behaviors. Figure 1 outlines the pathways we measured: empowerment, hope, communication of HIV status, and confidence in HIV status.

Empowered FSWs or those who are hopeful for their future might be more likely to negotiate condom use or be more selective about with whom they have sex [20, 26, 27]. Communication of HIV status between FSWs and their sexual partners might similarly effect FSWs’ condom use or their decision to engage in any sexual activity [28, 29]. Knowledge of HIV status, acquired through HIV (self-)testing, might further affect HIV risk-related sexual behaviors depending on FSWs’ concerns of HIV acquisition or transmission [30–34].

The potential pathways to sexual behavior change may differ between the two HIV self-testing delivery models, which provided differential motivation to visit healthcare facilities—i.e., participants in the direct provision arm did not have to visit a healthcare facility at all to test for HIV, while participants in the facility collection arm needed to visit a healthcare facility but did not need to engage with a healthcare provider to test for HIV. We selected variables from the 4-month assessment that captured the potential pathways from an HIV-self-testing offer to sexual behaviors.

To measure empowerment, we calculated the proportion of participants who reported always using a condom when they wanted during sexual intercourse with clients in the past month [21] and the proportion of participants who reported always or often asking clients to share their HIV status before engaging in sex (five-point Likert scale). To measure hope, we calculated the proportion of participants that are not likely depressed on the PHQ-9 depression diagnostic scale [35], a 0–27 point scale where scores ≥ 10 indicate the prevalence of likely depression [36]. To measure communication of HIV status we calculated the proportion of participants who reported always or often sharing their HIV status with clients before sex. To measure confidence in HIV status, participants were asked to report how likely it was, on a 1–10 ladder scale (10 being very likely), that they currently had HIV. If participants reported a 1 or a 10, we categorized them as confident in their HIV status. All pathway variables were self-reported and none were pre-specified for secondary analysis [25].

We also measured HIV testing mechanisms upstream of these pathways, including the proportion of FSWs who received/collected at least one HIV self-test kits, the proportion of FSWs who gave at an HIV self-test to others, the proportion of FSWs who tested for HIV since the start of the study.

### Statistical Analysis

All statistical models were intention-to-treat, complete case analyses conducted at the unit of the individual [37–39]. We estimated risk differences at 1 and 4 months for the sexual behaviors outcomes and at 4 months for the pathway variables using generalized linear regression models with a fixed effect for study arm and a random effect for peer educator. We reported mean differences for the count outcomes and percentage point (PP) differences for the binary outcomes. We chose linear regression models for our primary analyses because they generate risk differences, which are easier to interpret than risk ratios.

In addition to the primary analyses, we conducted three sensitivity analyses. First, we calculated incident risk ratios for the count outcomes and risk ratios for the binary outcomes using mixed-effects generalized linear models (Poisson distribution, log link, and robust standard error) with a fixed effect for study arm and random effect for peer education. We choose to use modified Poisson models, like those used in the primary trial paper [7, 40], over log-binomial models because they generate similar outcomes and converge more easily when study results are relatively common [40]. Second, we pooled the sexual behavior outcomes across the two HIV self-testing intervention arms and compared these pooled outcomes with those from the standard-of-care arm at 1 month and at 4 months using the generalized linear models that we used in the main analysis. Third, we conducted sub-group analyses where we explored the effect of the different HIV (self-) testing delivery models on sexual behavior outcomes among participants who self-reported testing HIV-negative and among participants who self-reported testing HIV-positive at their last HIV test at 1 month and at 4 months, again using the generalized linear models that we used in the main analysis.

We used Stata 13.1 (College Station, TX, USA) for all analyses. All statistical tests were two-sided with *p *< 0.05 considered statistically significant.

## Results

**Fig. 2:**
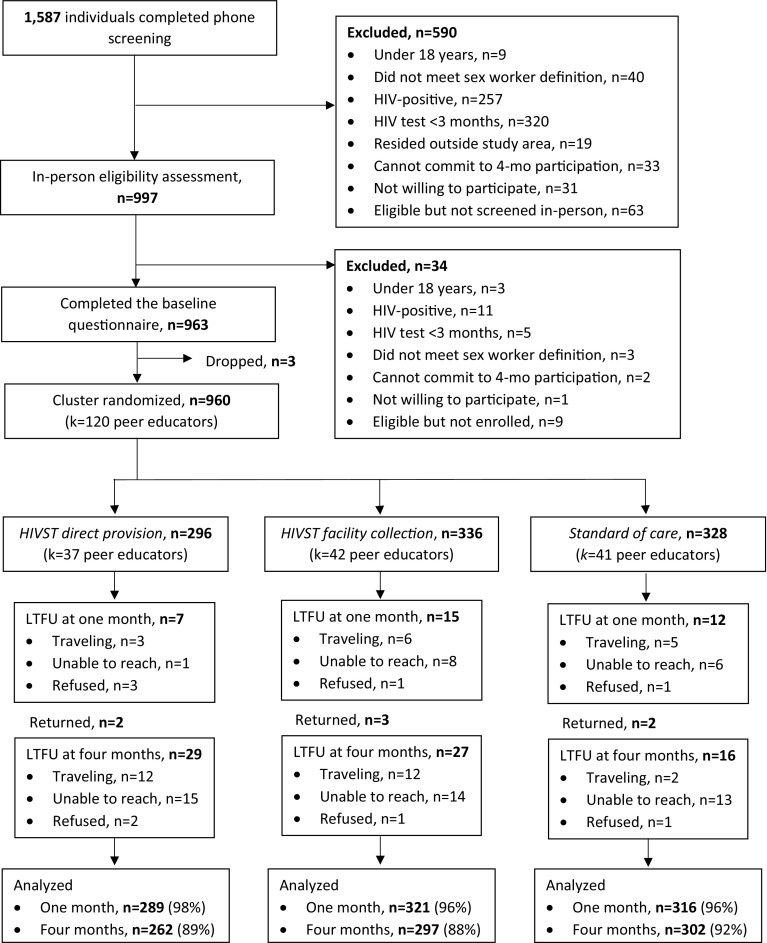
Participant recruitment, eligibility, randomization and follow-up. *HIVST* HIV self-test, *k* clusters, *LTFU* loss to follow-up, *mos* months

From October to November 2016, research assistants screened 1587 potential participants by phone and then 977 of these participants in-person. A total of 960 participants were enrolled and randomized: 296 (37 participant–peer educator groups) to the direct provision arm, 336 [42] to the facility collection arm, and 328 [41] to the standard-of-care arm (Fig. 2) [7]. At 1 month 96% of participant were retained in the study and at 4 months 90% of study participants were retained in the study.

The participants’ socio-demographic characteristics and self-reported sexual behaviors at baseline did not differ across study arms (Table 1). The median age of participants was 28 years [interquartile range (IQR) 24–32 years]. The majority of participants reported less than 9 years of education (53.8%), tested for HIV in the past 12 months (65.6%), and reported having non-client sexual partners (59.1%).

**Table 1:** Participants’ socio-demographic characteristics at baseline

	Direct provision (N = 296)	Facility collection (N = 336)	Standard-of-care (N = 328)	Total (N = 960)
Characteristics				
Age (med, IQR)	28 (24 to 32)	28 (25 to 32)	28 (24 to 32)	28 (24 to 32)
Education				
No formal	24 (8.1%)	35 (10.4%)	20 (6.1%)	79 (8.2%)
Primary/junior	121 (40.9%)	155 (46.1%)	161 (49.1%)	437 (45.5%)
Secondary	143 (48.3%)	136 (40.5%)	144 (43.9%)	423 (44.1%)
Vocational	2 (0.7%)	6 (1.8%)	0	8 (0.8%)
Tertiary	6 (2.0%)	4 (1.2%)	3 (1.0%)	13 (1.4%)
Monthly income, USD^a^				
No income	4 (1.4%)	0	1 (0.3%)	5 (0.5%)
< $35.67	63 (21.3%)	76 (22.9%)	51 (15.6%)	190 (19.9%)
$35.67–$74.32	90 (30.4%)	117 (35.2%)	125 (38.3%)	332 (34.8%)
$74.32–$148.64	104 (35.1%)	107 (32.2%)	117 (35.9%)	328 (34.4%)
> $148.64	35 (11.9%)	32 (9.6%)	32 (9.8%)	99 (10.4%)
Timing of last HIV test				
> 3–6 months	123 (37.5%)	119 (35.6%)	108 (36.7%)	350 (36.6%)
> 6–12 months	102 (31.1%)	88 (26.4%)	90 (30.6%)	280 (19.3%)
> 12–24 months	42 (12.8%)	68 (20.4%)	46 (15.7%)	156 (16.3%)
> 24 months	42 (12.8%)	42 (12.6%)	30 (10.2%)	114 (11.9%)
Never tested	19 (5.8%)	17 (5.1%)	20 (6.8%)	56 (5.9%)
Intimate partner violence, past 12 months				
Physical	102 (34.5%)	132 (39.3%)	115 (35.3%)	349 (36.4%)
Sexual	89 (30.1%)	105 (31.3%)	94 (28.8%)	288 (30.1%)
Price for vaginal sex, USD (mean, sd)				
With a condom	$3.77 ($3.99)	$2.98 ($2.74)	$3.04 ($3.13)	$3.24 ($3.31)
Without a condom	$10.14 ($10.36)	$8.40 ($6.73)	$11.58 ($16.01)	$9.94 ($11.46)
Have non-client partners	120 (61.5%)	125 (57.1%)	133 (58.9%)	378 (59.1%)
Average number of clients/night (mean, sd)	5.7 (3.1)	5.9 (3.4)	6.1 (4.6)	5.9 (3.8)
Inconsistent condom use with clients^b^	125 (42.5%)	141 (42.2%)	122 (37.4%)	388 (40.7%)
Number of non-clients, past month (mean, sd)	1.8 (2.7)	1.6 (2.3)	1.6 (2.8)	1.6 (2.6)
Inconsistent condom use with non-clients^c^	157 (54.9%)	167 (51.5%)	165 (51.2%)	489 (52.5%)

*N* total number of participants, *med* median, *IQR* interquartile range, *sd* standard deviation

^a^Price categories in US dollars (USD), 10^th^ October, 2016 exchange rate (1 USD = 3363.85 Ugandan Shillings)

^b^Defined as not using a condom with at least one client on an average working night

^c^Defined as never using a condom with at least one non-client sexual partner in the past month

**Fig. 3:**
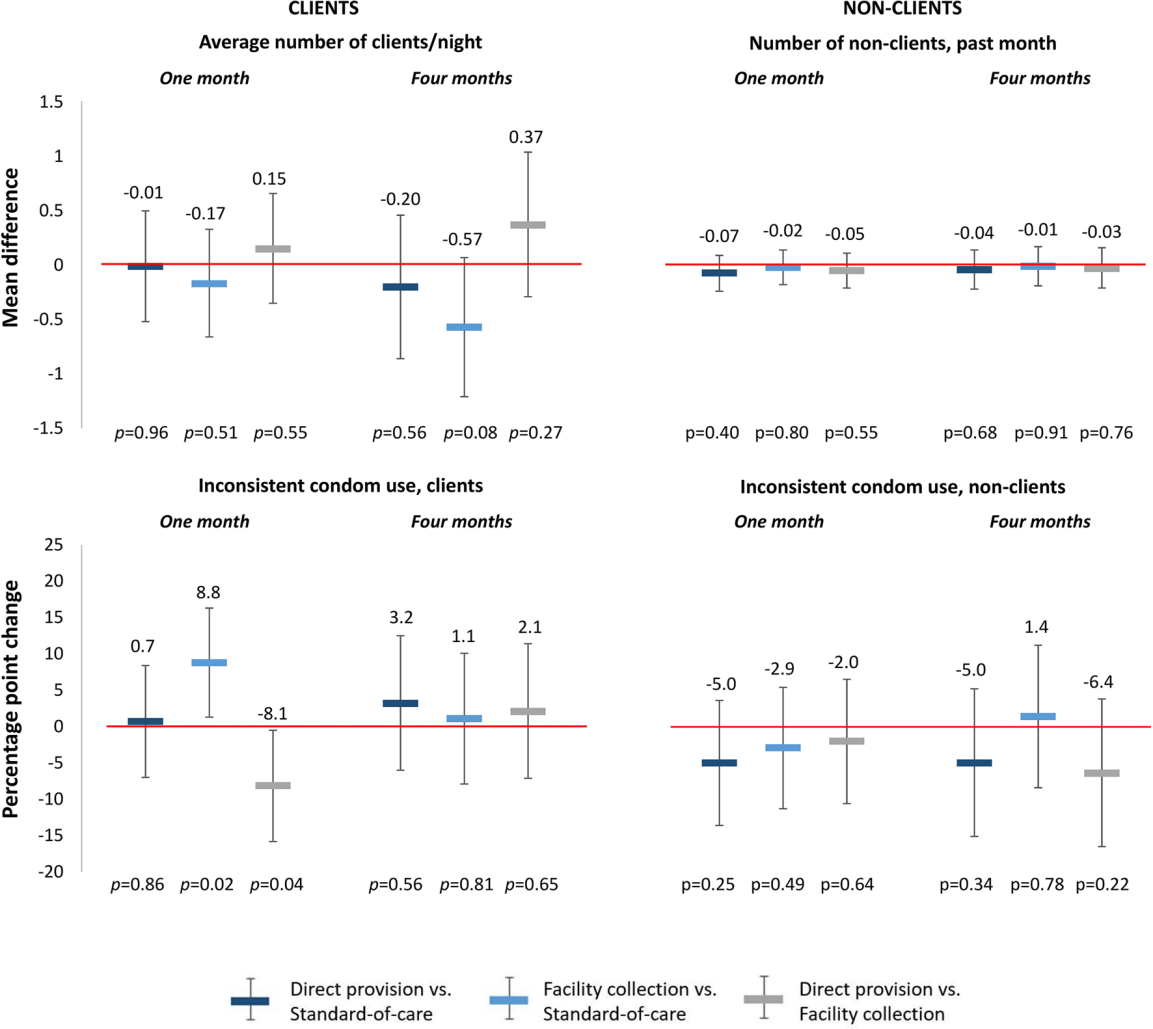
The effect of the different HIV self-testing delivery models on FSWs’ sexual behaviors with client and non-client sexual partners at 1 month and 4 months. Inconsistent condom use with clients was defined as not using a condom with at least one client on an average working night. Inconsistent condom use with non-clients was defined as never using a condom with at least one non-client sexual partner in the past month. Comparisons between study arms: direct provision versus standard-of-care (dark blue), facility collection versus standard-of-care (light blue), direct provision versus facility collection (gray) (Color figure online)

Participants’ sexual behaviors by study arm at 1 month and at 4 months are shown in Table 2, while the effect size estimates (mean differences and PP changes) are shown in Fig. 3. The different HIV self-testing delivery models did not affect FSWs’ average number of *clients* per night at 1 month and at 4 months, nor did they affect FSWs’ inconsistent condom use with *clients* at 4 months. At 1 month, participants in the HIV self-testing facility collection arm were 8.8 PPs more likely to inconsistently use condoms with clients (95% CI 1.3 to 16.3, *p *= 0.02) compared to those in the standard-of-care arm, but there were no statistically significant differences in this outcome among participants in the HIV self-testing facility collection arm compared to the standard-of-care arm at 4 months (PP 1.1, 95% CI − 7.9 to 10.1, *p *= 0.81).

**Table 2:** Participants’ sexual behaviors by study arm at 1 month and at 4 months

	One month			Four months		
Outcomes	Direct provision	Facility collection	Standard-of-care	Direct provision	Facility collection	Standard-of-care
Clients						
Average number clients/night (mean, sd)	5.5 (3.1)	5.3 (2.8)	5.6 (4.2)	5.6 (3.4)	5.3 (3.1)	5.9 (4.0)
Inconsistent condom use^a^ (*n*/*N*, %)	60/287 (20.9)	93/321 (29.0)	57/314 (18.2)	87/262 (33.2)	95/295 (32.2)	82/299 (27.4)
Non-clients						
Number non-clients, past month (mean, sd)	0.8 (0.8)	0.8 (0.9)	0.9 (0.9)	0.8 (0.9)	0.8 (0.9)	0.9 (0.9)
Inconsistent condom use^b^ (*n*/*N*, %)	129/286 (45.1)	146/313 (46.7)	153/310 (49.4)	124/261 (47.5)	155/297 (52.2)	156/300 (52.0)

*n* number of participants reporting outcome, *N* total number of participants, *sd* standard deviation

^a^Defined as not using a condom with at least one client on an average working night

^b^Defined as never using a condom with at least one non-client sexual partner in the past month

The different HIV self-testing delivery models did not affect FSWs’ number of *non*-*client* sexual partners in the past month nor their inconsistent condom use with *non*-*client* sexual partners at 1 month and at 4 months (Table 2; Fig. 3).

Table 3 describes the HIV self-testing mechanisms and the variables associated with potential pathways through which HIV self-testing might affect FSWs’ sexual behaviors. At 4 months, almost all participants in the HIV intervention arms received at least one HIV self-test, only a few participants gave an HIV self-test to a client, friend, or family member, and the vast majority of study participants across the study arms tested for HIV. As reported in Ortblad et al., the participants in HIV self-testing intervention arms were significantly more likely to test for HIV than those in the standard-of-care arm [7].

**Table 3:** Pathways through with HIV self-testing might affect FSWs’ sexual behaviors at 4 months

Outcomes	Percentage of participants			Effect size estimates					
	Direct provision	Facility collection	Standard-of-care	Direct provision versus Standard-of-care		Facility collection versus Standard-of-care		Direct provision versus facility collection	
				PP^a^ (95% CI)	*p*	PP^a^ (95% CI)	*p*	PP^a^ (95% CI)	*p*
Mechanisms									
Received/collected HIV self-test^b^	260/262 (99.2%)	283/297 (95.3%)	n/a	n/a		n/a		3.8 (1.1 to 6.5)	0.01
Gave an HIV self-test to others^c^	4/262 (1.5%)	1/297 (0.3%)	n/a	n/a		n/a		1.2 (− 0.1 to 2.5)	0.07
Tested for HIV^d^	261/262 (99.6%)	288/297 (97.0%)	263/302 (87.1%)	12.9 (7.6 to 18.2)	< 0.001	10.3 (5.2 to 15.4)	< 0.001	2.6 (− 2.7 to 7.8)	0.34
Pathway variables									
Empowerment									
Always used a condom with clients when want to^e^	203/262 (77.5%)	226/297 (76.1%)	225/301 (74.8%)	2.7 (− 6.0 to 11.3)	0.55	1.4 (− 7.0 to 9.7)	0.75	1.3 (− 7.3 to 9.9)	0.77
Always/often ask client to share HIV status	16/262 (6.1%)	17/297 (5.7%)	21/302 (7.0%)	− 0.7 (− 5.6 to 4.3)	0.79	− 1.2 (− 6.0 to 3.6)	0.63	0.5 (− 4.4 to 5.4)	0.84
Hope									
Not depressed (PHQ-9)^f^	208/262 (79.4%)	214/297 (72.1%)	206/302 (68.2%)	10.3 (− 0.1 to 20.7)	0.05	4.0 (− 6.0 to 14.1)	0.43	6.3 (− 4.1 to 16.6)	0.24
Communication of HIV status									
Always/often share HIV status with client	30/260 (11.5%)	20/296 (6.8%)	22/300 (7.3%)	4.3 (− 2.3 to 11.0)	0.20	− 0.7 (− 7.2 to 5.7)	0.82	5.1 (− 1.6 to 11.7)	0.14
Confidence in HIV knowledge									
Confident in HIV status^g^	126/262 (48.1%)	132/297 (44.4%)	148/302 (49.0%)	− 1.4 (− 13.8 to 11.1)	0.83	− 4.6 (− 16.6 to 7.5)	0.46	3.2 (− 9.2 to 15.6)	0.61

*PP* percentage point change, *CI* confidence interval, *p p* value

^a^Linear models with study arm fixed effects and peer educator random effects

^b^Participants reported receiving or collecting at least one HIV self-test from their peer educator or the healthcare facility

^c^Participants reported not using at least one of their HIV self-tests to test themselves and reported giving the self-test to a client, friend, or family member

^d^Any HIV testing reported since the start of the study (Ortblad et al. PLoS Med [7])

^e^Participants were asked if there was a time in the past month when they wanted to use a condom during sexual intercourse with a client and did not

^f^Participants were categorized as not depressed (scores < 10) using the PHQ-9 scale (0–27 points)

^g^Participants report likelihood that they currently have HIV as 1 (very unlikely) or 10 (very likely) on a 1–10 ladder

There were largely no statistically significant differences across study arms in any of the potential pathway variables: the proportion participants who of reported always using a condom with clients when they wanted and always or often asked clients to share their HIV status (empowerment), the proportion participants who were categorized as not likely depressed (hope), the proportion participants who reported always or often sharing their HIV status with clients (HIV status communication), and the proportion participants who were categorized as confident in their HIV status (confidence in HIV status). While more participants reported not being depressed in the HIV self-testing direct provision arm compared to the standard-of-care arm at 4 months (PP 10.3, 95% CI − 0.1 to 20.7, *p *= 0.05), this was only of borderline significance.

In our sensitivity analyses measuring risk ratios instead of risk differences, HIV self-testing did not significantly affect FSWs' sexual behaviors at 1 month and at 4 months (Appendix Table 4). Similarly, when we pooled sexual behaviors outcomes across the HIV self-testing arms and compared outcomes from this pooled arm with those from the standard-of-care arm, HIV self-testing did not significantly affect sexual behaviors at 1 months and at 4 months (Appendix Tables 5, 6).

In the sub-group analyses among participants who self-reported testing *HIV*-*negative* and among those who self-reported testing *HIV*-*positive* at their last test, the different HIV self-testing delivery models largely had no effect on FSWs’ sexual behaviors at 1 month and at 4 months compared to referral to standard HIV testing services (Appendix Tables 7, 8). The only statistically significant difference was among participants who self-reported testing *HIV-positive *in the direct provision HIV self-testing arm. At 1 month, inconsistent condom use with *clients* was significantly lower among these participants compared to participants who self-reported testing *HIV-positive *in the facility collection arm (PP − 17.5, 95% CI − 33.8 to − 1.2, *p *= 0.04), but these differences did not persist at 4 months (Appendix Table 8).

Two adverse events related to HIV self-testing were reported over the duration of the trial: intimate partner violence following discovery of an HIV self-test, and mental distress from a perceived HIV-positive self-test result (the participant tested HIV-negative at a healthcare facility) [7].

## Discussion

We tested the causal effects of two HIV self-testing delivery models on FSWs’ sexual risk-taking. Neither the direct delivery of HIV self-tests nor the facility collection of HIV self-tests affected FSWs’ sexual behaviors with client and non-client sexual partners compared to referral to standard-of-care HIV testing services in urban Uganda. Our study adds to the emerging evidence on the effects of HIV self-testing on sexual behaviors [41–43].

Understanding the effect of HIV self-testing—delivered by different approaches—on behaviors directly related to the acquisition and transmission of HIV is important so that we understand the full implications of this testing technology. This understanding takes on particular significance as government in several sub-Saharan African countries are implementing or considering national scale-up of HIV self-testing as one routine testing option [23]. Our findings demonstrate no increase in sexual risk-taking among FSWs in response to the delivery of HIV self-tests. Routine policies for HIV self-testing are likely a safe component of comprehensive HIV testing and prevention strategies for FSWs. As HIV self-testing is scaled up to the national level, accompanying research should identify approaches to deliver HIV self-testing in ways that reduce sexual risk and increase access to other HIV interventions, including HIV pre-exposure prophylaxis and treatment-as-prevention.

Unlike the prior studies on this topic—two among men who have sex with men (MSM) in high-incomes settings [17, 18] and one among Zambian FSWs [19]—this study did not find that delivery of HIV self-testing *reduced* FSWs’ HIV sexual risk-taking behaviors. It is immediately obvious that FSWs in Uganda are a very different population from MSM in high-income countries [44, 45] and might thus react differently to receiving HIV self-tests. For instance, MSM may have more power over their sex lives than FSWs in sub-Saharan Africa [46, 47], who may not be able to act on changes in their ability to test for HIV and the information resulting from HIV testing.

It is less obvious why our findings here differ from those in the study among FSWs in Zambian transit towns. However, there is significant variation in FSW populations globally in regards to the locations in which they work and live, their type of clientele, and their risk of HIV acquisition [48, 49]. For instance, compared to the FSWs in the Zambian study, the FSWs in this study worked in more urban environments and consequently reported a greater number of clients on an average working night [6, 7]. As a result, the FSWs in our study might have had more casual versus steady clients compared to the FSWs in Zambia and this may have affected their ability to negotiate condom use. The risk of HIV transmission per commercial sex act is also greater in Zambia compared to Uganda as a result of higher population HIV prevalence [22, 50]. The FSWs in Zambia may thus have been more concerned about acquiring HIV compared to those in Uganda, and thus engaged in differential HIV sexual risk-taking behaviors as a result of HIV self-testing.

Jointly with this previous study, however, our study in a very different FSW population supports the policy recommendation that HIV self-testing is safe regarding its effects on sexual behavior. These studies suggest that concerns about HIV self-testing leading to increased sexual risking taking are unlikely to be true and thus should not be a reason for failing to make HIV self-tests available to FSWs in sub-Saharan Africa.

None of the pathways through which we hypothesized that the delivery of HIV self-tests might affect FSWs’ sexual behaviors were affected by the HIV self-testing interventions, which may further explain why we found no differences in FSWs’ HIV risk-related sexual behaviors by study arm. Even though significantly more FSWs in the HIV self-testing intervention arms tested for HIV since the start of the study [7], there were no differences in FSWs’ empowerment, hope, HIV status communication, and knowledge of HIV status across study arms. The delivery of HIV self-tests may not have affected these pathway variables because FSWs may have little power within their sexual relationships with clients to negotiate the use of condom [46, 47].

Additionally, the prevalence of sexual and intimate partner violence among FSWs in this study was high and FSWs’ economic incentives for the provision of condomless sex were large. HIV self-testing may not have changed FSWs’ knowledge of HIV status in this study because the majority of participants had tested for HIV in the past year at baseline and thus may already have had a good idea of their HIV status. It is also possible that the FSWs who HIV self-tested may not have believed the result of a test that used oral-fluid instead of blood or may not have known how to interpret the self-test result [51].

Our study has a number of strengths. It is one of a few studies to explore the effect of HIV self-testing delivery models on sexual risk-taking behaviors [17–19] and focuses on a population that is a priority for HIV prevention interventions. Our study further has causal strength, because it is a randomized controlled trial. Additionally, the study outlines and explores different pathways through which HIV self-testing delivery models might affect FSWs’ sexual behaviors.

Our study also has several limitations. First, all sexual behaviors outcomes were self-reported by participants and subject to recall and social desirability bias, a common limitation of most studies of sexual behavior. Direct measures of sexual behavior are not feasible and biomarkers for sexual activity are still not established for routine population-based application because their performance is poor and they are expensive and difficult to collect [52, 53]. In the absence of direct or biological measures, self-reported sexual behavior data is the best available option, despite its obvious shortcomings.

Second, the follow-up period of the trial, 4 months, was a relatively short duration; preventing us from measuring the longer term effects of HIV self-testing delivery models on FSWs’ sexual behaviors. Third, the trial was not specifically powered to measure differences in sexual behaviors outcomes across study arms, contributing to greater uncertainty around the effect size estimates, especially in the HIV status sub-group analyses. All of our effect size estimates, however, were close to zero and had narrow confidence intervals, suggesting the null effect of HIV self-testing delivery models on FSWs’ sexual behaviors. Fourth, we only collected measurements of the pathway variables at the end of the study (4 months) and thus were unable to assess how the intervention effected these variables at other time points.

Finally, due to the diversity of FSW populations, the generalizability of these results might be limited in settings outside urban Uganda. But taken together with the previous evidence in a very different FSW population in Zambian transit towns, a robust conclusion appears to be emerging: HIV self-testing—delivered by different approaches and to different FSW populations—does not increase sexual risk taking.

## Conclusion

We find that HIV self-testing does not increase sexual risk-taking among FSWs. Routine policies for HIV self-testing are thus likely safe for this key population in the HIV response. As national governments in sub-Saharan Africa scale up HIV self-testing, accompanying research should identify approaches to deliver HIV self-testing in ways that reduce sexual risk and increase access to other HIV interventions.

## Appendix

See Tables 4, 5, 6, 7 and 8.

**Table 4:** Sensitivity analysis 1: incident risk ratios and risk ratios of HIV self-testing on risky sexual behaviors among participants self-reporting HIV-negative status at 1 month and 4 months

Outcomes	Direct provision versus Standard-of-care		Facility collection versus Standard-of-care		Direct provision versus facility collection	
	Risk ratio^a^ (95% CI)	*p*	Risk ratio^a^ (95% CI)	*p*	Risk ratio^a^ (95% CI)	*p*
One month						
Clients						
Avg # clients/night (IRR)	0.98 (0.85 to 1.23)	0.74	0.97 (0.85 to 1.10)	0.62	1.01 (0.89 to 1.15)	0.89
Inconsistent condom use^b^ (RR)	1.15 (0.69 to 1.90)	0.59	1.64 (1.05 to 2.54)	0.03	0.70 (0.44 to 1.12)	0.14
Non-clients						
# Non-clients, past month (IRR)	0.93 (0.74 to 1.17)	0.55	0.96 (0.76 to 1.21)	0.73	0.97 (0.76 to 1.24)	0.82
Inconsistent condom use^c^ (RR)	0.91 (0.74 to 1.13)	0.40	0.95 (0.78 to 1.15)	0.57	0.97 (0.77 to 1.21)	0.77
Four months						
Clients						
Avg # clients/night (IRR)	0.93 (0.79 to 1.10)	0.40	0.89 (0.78 to 1.03)	0.12	1.04 (0.90 to 1.21)	0.58
Inconsistent condom use^b^ (RR)	1.20 (0.80 to 1.80)	0.37	1.16 (0.78 to 1.72)	0.46	1.04 (0.69 to 1.56)	0.87
Non-clients						
# Non-clients, past month (IRR)	0.94 (0.73 to 1.22)	0.64	0.99 (0.80 to 1.23)	0.95	0.95 (0.73 to 1.22)	0.68
Inconsistent condom use^c^ (RR)	0.91 (0.72 to 1.16)	0.44	1.00 (0.81 to 1.23)	1.00	0.91 (0.71 to 1.16)	0.45

*RR* risk ratio, *IRR* incidence risk ratio, *CI* confidence interval, *p* p-value

^a^Linear models with study arm fixed effects and peer educator random effects, adjusted for sexual behavior at baseline

^b^Defined as not using a condom with at least one client on an average working night

^c^Defined as never using a condom with at least one non-client sexual partner in the past month

**Table 5:** Sensitivity analysis 2: risk differences of pooled HIV self-testing arms on risky sexual behaviors at 1 and 4 months

	*n*/*N* (%) or mean (sd)			
Outcomes	Pooled HIVST arms	Standard-of-care	Risk differences^a^ (95% CI)	*p*
One month				
Clients				
Avg # clients/night (mean, sd; MD)	5.4 (2.9)	5.6 (4.2)	− 0.09 (− 0.53 to 0.34)	0.67
Inconsistent condom use^b^ (*n*/*N*; PP)	153/608 (25.2%)	57/314 (18.2%)	5.0 (− 1.7 to 11.7)	0.14
Non-clients				
# Non-clients, past month (mean, sd; MD)	0.8 (0.9)	0.9 (0.9)	− 0.04 (− 0.18 to 0.10)	0.53
Inconsistent condom use^c^ (*n*/*N*; PP)	275/599 (45.9%)	153/310 (49.4%)	− 3.9 (− 11.2 to 3.4)	0.29
Four months				
Clients				
Avg # clients/night (mean, sd; MD)	5.4 (3.2)	5.9 (3.4)	− 0.40 (− 0.96 to 0.17)	0.17
Inconsistent condom use^b^ (*n*/*N*; PP)	182/557 (32.7%)	82/299 (27.4%)	2.1 (− 5.8 to 9.9)	0.60
Non-clients				
# Non-clients, past month (mean, sd; MD)	0.8 (0.9)	0.9 (0.9)	− 0.02 (− 0.18 to 0.13)	0.77
Inconsistent condom use^c^ (*n*/*N*; PP)	279/558 (50.0%)	156/300 (52.0%)	− 1.6 (− 10.2 to 7.0)	0.72

*HIVST* HIV self-testing, *n* number of participants reporting outcome, *N* total number of participants, *MD* mean difference, *PP* percentage point change, *CI* confidence interval, *p* p-value

^a^Linear models with study arm fixed effects and peer educator random effects, adjusted for sexual behavior at baseline

^b^Defined as not using a condom with at least one client on an average working night

^c^Defined as never using a condom with at least one non-client sexual partner in the past month

**Table 6:** Sensitivity analysis 2: participants’ sexual behaviors by study arm by self-reported HIV status at 1 month and at 4 months

Outcomes	One month			Four months		
	Direct provision	Facility collection	Standard-of-care	Direct provision	Facility collection	Standard-of-care
Participants, HIV-negative^a^						
Clients						
Avg # clients/night (mean, sd)	5.5 (3.1)	5.2 (2.7)	5.6 (4.4)	5.5 (3.2)	5.1 (2.7)	5.8 (3.9)
Inconsistent condom use^b^	50/239 (20.9%)	59/247 (23.9%)	46/262 (17.6%)	68/212 (32.1%)	60/207 (29.0%)	68/245 (27.8%)
Non-clients						
# Non-clients, past month (mean, sd)	0.8 (0.8)	0.8 (0.9)	0.9 (0.9)	0.9 (0.9)	0.8 (0.7)	0.9 (1.0)
Inconsistent condom use^c^	115/238 (48.3%)	113/243 (46.5%)	127/258 (49.2%)	105/211 (49.8%)	104/208 (50.0%)	129/245 (52.7%)
Participants, HIV-positive^a^						
Clients						
Avg # clients/night (mean, sd)	5.5 (2.9)	5.8 (2.9)	6.4 (3.6)	6.6 (4.8)	5.6 (3.7)	6.8 (4.2)
Inconsistent condom use^b^	7/38 (18.4%)	26/54 (48.2%)	9/38 (23.7%)	15/34 (44.1%)	26/65 (40.0%)	11/43 (25.6%)
Non-clients						
# Non-clients, past month (mean, sd)	0.6 (0.7)	0.9 (1.0)	0.9 (0.8)	0.5 (0.8)	1.1 (1.1)	0.6 (0.6)
Inconsistent condom use^c^	10/38 (26.3%)	24/51 (47.1%)	19/37 (51.4%)	11/34 (32.4%)	36/66 (54.6%)	20/44 (45.5%)

*avg* average, *sd* standard deviation, mos months

^a^Self-reported results of last HIV test

^b^Defined as not using a condom with at least one client on an average working night

^c^Defined as never using a condom with at least one non-client sexual partner in the past month

**Table 7:** Sensitivity analysis 3: risk differences of HIV self-testing on risky sexual behaviors among participants self-reporting HIV-negative status at 1 and 4 months

Outcomes	Direct provision versus standard-of-care		Facility collection versus standard-of-care		Direct provision versus facility collection	
	Risk difference^a^ (95% CI)	*p*	Risk difference^a^ (95% CI)	*p*	Risk difference^a^ (95% CI)	*p*
One month						
Clients						
Avg # clients/night (MD)	− 0.06 (− 0.46 to 0.57)	0.83	− 0.21 (− 0.72 to 0.29)	0.41	0.27 (− 0.25 to 0.79)	0.31
Inconsistent condom use^b^ (PP)	2.1 (− 5.7 to 10.0)	0.60	6.1 (− 1.7 to 13.9)	0.12	− 4.0 (− 11.9 to 4.0)	0.33
Non-clients						
# Non-clients, past month (MD)	− 0.05 (− 0.23 to 0.13)	0.57	− 0.02 (− 0.20 to 0.16)	0.81	− 0.03 (− 0.21 to 0.15)	0.74
Inconsistent condom use^c^ (PP)	− 3.7 (− 13.1 to 5.7)	0.44	− 3.1 (− 12.4 to 6.2)	0.51	− 0.6 (− 10.1 to 8.9)	0.90
Four months						
Clients						
Avg # clients/night (MD)	− 0.26 (− 0.95 to 0.44)	0.47	− 0.69 (− 1.38 to 0.00)	0.05	0.43 (− 0.28 to 1.15)	0.23
Inconsistent condom use^b^ (PP)	1.3 (− 8.1 to 10.7)	0.79	− 0.2 (− 9.6 to 9.1)	0.96	1.5 (− 8.1 to 11.1)	0.75
Non-clients						
# Non-clients, past month (MD)	− 0.04 (− 0.22 to 0.15)	0.70	− 0.11 (− 0.30 to 0.08)	0.24	0.07 (− 0.12 to 0.27)	0.45
Inconsistent condom use^c^ (PP)	− 5.7 (− 16.3 to 5.0)	0.30	− 1.2 (− 11.8 to 9.4)	0.82	− 4.5 (− 15.5 to 6.5)	0.42

*MD* mean difference, *PP* percentage point difference, *CI* confidence interval, *p* p-value

^a^Linear models with study arm fixed effects and peer educator random effects, adjusted for sexual behavior at baseline

^b^Defined as not using a condom with at least one client on an average working night

^c^Defined as never using a condom with at least one non-client sexual partner in the past month

**Table 8:** Sensitivity analysis 3: risk differences of HIV self-testing on risky sexual behaviors among participants self-reporting HIV-positive status at 1 and 4 months

Outcomes	Direct provision versus standard-of-care		Facility collection versus standard-of-care		Direct provision versus facility collection	
	Risk differences^a^ (95% CI)	*p*	Risk differences^a^ (95% CI)	*p*	Risk differences^a^ (95% CI)	*p*
One month						
Clients						
Avg # clients/night (MD)	− 0.73 (− 2.05 to 0.58)	0.28	− 0.34 (− 1.61 to 0.94)	0.61	− 0.40 (− 1.67 to 0.88)	0.54
Inconsistent condom use^b^ (PP)	− 7.9 (− 25.2 to 9.4)	0.37	9.6 (− 6.9 to 26.2)	0.25	− 17.5 (− 33.8 to − 1.2)	0.04
Non-clients						
# Non-clients, past month (MD)	− 0.22 (− 0.59 to 0.16)	0.26	− 0.03 (− 0.38 to 0.32)	0.87	− 0.19 (− 0.55 to 0.17)	0.31
Inconsistent condom use^c^ (PP)	− 17.8 (− 40.2 to − 4.7)	0.12	− 4.8 (− 25.8 to 16.1)	0.65	− 12.9 (− 34.2 to 8.3)	0.23
Four months						
Clients						
Avg # clients/night (MD)	0.42 (− 1.15 to 1.99)	0.60	− 0.53 (− 1.86 to 0.81)	0.44	0.95 (− 0.52 to 2.42)	0.21
Inconsistent condom use^b^ (PP)	15.8 (− 3.4 to 35.1)	0.11	3.5 (− 13.6 to 20.6)	0.69	12.3 (− 5.7 to 30.4)	0.18
Non-clients						
# Non-clients, past month (MD)	− 0.09 (− 0.52 to 0.34)	0.68	0.27 (− 0.10 to 0.65)	0.16	− 0.36 (− 0.77 to − 0.04)	0.08
Inconsistent condom use^c^ (PP)	− 4.8 (− 28.3 to 18.7)	0.69	7.6 (− 12.8 to 27.9)	0.47	− 12.4 (− 34.5 to 9.7)	0.27

*MD* mean difference, *PP* percentage point difference, *CI* confidence interval, *p* p-value

^a^Linear models with study arm fixed effects and peer educator random effects, adjusted for sexual behavior at baseline

^b^Defined as not using a condom with at least one client on an average working night

^c^Defined as never using a condom with at least one non-client sexual partner in the past month
